# Using RE-AIM to examine implementation of a tele-nephrology program for veterans living in rural areas

**DOI:** 10.3389/frhs.2023.1205951

**Published:** 2023-09-13

**Authors:** Kristin M. Mattocks, Aimee Kroll-Desrosiers, Susan Crowley, Katherine Tuozzo, Ian Rifkin, David Moore, Lorrie Walker, Ramon Bonegio

**Affiliations:** ^1^ VA Central Western Massachusetts Healthcare System, Leeds, MA, United States; ^2^ Population and Quantitative Health Sciences, University of Massachusetts Chan Medical School, Worcester, MA, United States; ^3^ VA Connecticut Healthcare System, West Haven, CT, United States; ^4^ Yale University School of Medicine, New Haven, CT, United States; ^5^ VA Boston Healthcare System, Boston, MA, United States; ^6^ Boston University School of Medicine, Boston, MA, United States

**Keywords:** veterans, kidney disease, access to care, health services, telemedicine

## Abstract

**Introduction:**

Chronic kidney disease (CKD) and refractory hypertension (rHTN) are common, chronic conditions that affect 10%–16% of Veterans. Several small studies have suggested that tele-nephrology can deliver nephrology care effectively to rural Veterans. The purpose of this evaluation was to examine perceptions and experiences with this tele-nephrology program among spoke site staff and clinicians using the Reach, Effectiveness, Adoption, Implementation, and Maintenance (RE-AIM) framework to guide our understanding of tele-nephrology implementation.

**Methods:**

We conducted semi-structured interviews with fourteen clinicians at five tele-nephrology spoke sites. We used content analysis to analyze the results using our RE-AIM framework.

**Results:**

Five major themes arose: (1) Active engagement of a centralized clinical champion was a key factor in early success of tele-nephrology program; (2) Transition from community-based nephrology to VA tele-nephrology was heralded as the most meaningful indicator of the effectiveness of the intervention; (3) Effective adoption strategies included bi-weekly training with Hub nephrology staff and engagement of a local renal champion; (4) Meeting the needs of Veterans through proper staffing during tele-nephrology examinations was a key priority in facility program implementation; and (5) Growing reliance on Hub nephrologists may give rise to insufficient availability of nephrology appointments in some Spoke sites.

**Discussion:**

This evaluation represents an important step forward as VA considers how to provide care to Veterans at facilities without VA specialty providers. The COVID-19 pandemic has drastically shifted options for Veterans, and increasingly, the VA is moving to shift care from community to VA via virtual care. Further research should examine how the VA manages potential problems related to access to virtual providers and examine Veteran perspectives on community in-person vs. virtual VA care.

## Introduction

Chronic kidney disease (CKD) and refractory hypertension (rHTN) are common, chronic conditions that are often associated and are best managed by a nephrologist. CKD and rHTN affect 10%–16% and 9%–13% of Veterans respectively (1, 2). Disease prevalence increases with age and is higher in Veterans with diabetes and hypertension (2). Veterans living in rural and highly rural areas have a high prevalence of CKD and rHTN but are less likely to receive specialist nephrology care. Nephrology care delivered to rural veterans is also frequently of lower quality (3, 4). Rural Veterans with CKD or rHTN have a higher mortality and are hospitalized more frequently than Veterans living in urban or suburban areas (4).

Veterans with CKD or rHTN benefit from seeing a specialist nephrologist and this is especially true for those with both CKD and diabetes (5–7); however, only 38% of Veterans with CKD received specialty care from a nephrologist prior to developing kidney failure (4). Pre-dialysis nephrology care reduced the risk of death by 12% during 2.9 years of follow-up (4). As a result of these data, the Veterans' Health Administration (VHA) directive 1,053 (CKD Prevention and Management) requires primary care providers to screen patients for kidney disease and refer them for specialist nephrology care early in the course of their illness (8).

Veterans with CKD and rHTN who attend large VA Medical Centers that are usually referred to a nephrology team for specialist care. In contrast, Veterans who attend smaller VA facilities and especially those living in rural areas. do not have access to VA nephrologists, and must receive their nephrology care from community providers. The Veterans Access, Choice, and Accountability (Choice) Act of 2014 and its successor, the VA Maintaining Systems and Strengthening Integrated Outside Networks (MISSION) Act of 2018, dramatically increased Veterans' ability to seek care in their communities, at VA expense, if they meet certain access criteria. Under the MISSION Act, if Veterans are unable to receive specialty care within 28 days or have to travel more than 60 min to get such care, they can qualify for receiving care in the community. Since the MISSION Act was implemented, nearly 2.5 million Veterans have been authorized to receive community care.

Approximately 5 million Veterans (24.1%) reside in rural areas of the USA (9). These rural Veterans struggle to access specialist nephrology care. VA-nephrology services are often located in large urban centers with academic affiliates while rural facilities either offer no VA-nephrology care or depend on a single provider to cover large geographic areas (10, 11). Rural areas are also lack non-VA community nephrologist and access is limited by long drive times and long wait times to get an appointment. Several small studies have suggested that tele-nephrology can deliver nephrology care effectively to rural Veterans and this achieves outcomes comparable to that of usual specialist care (10, 11).

**Figure 1:**
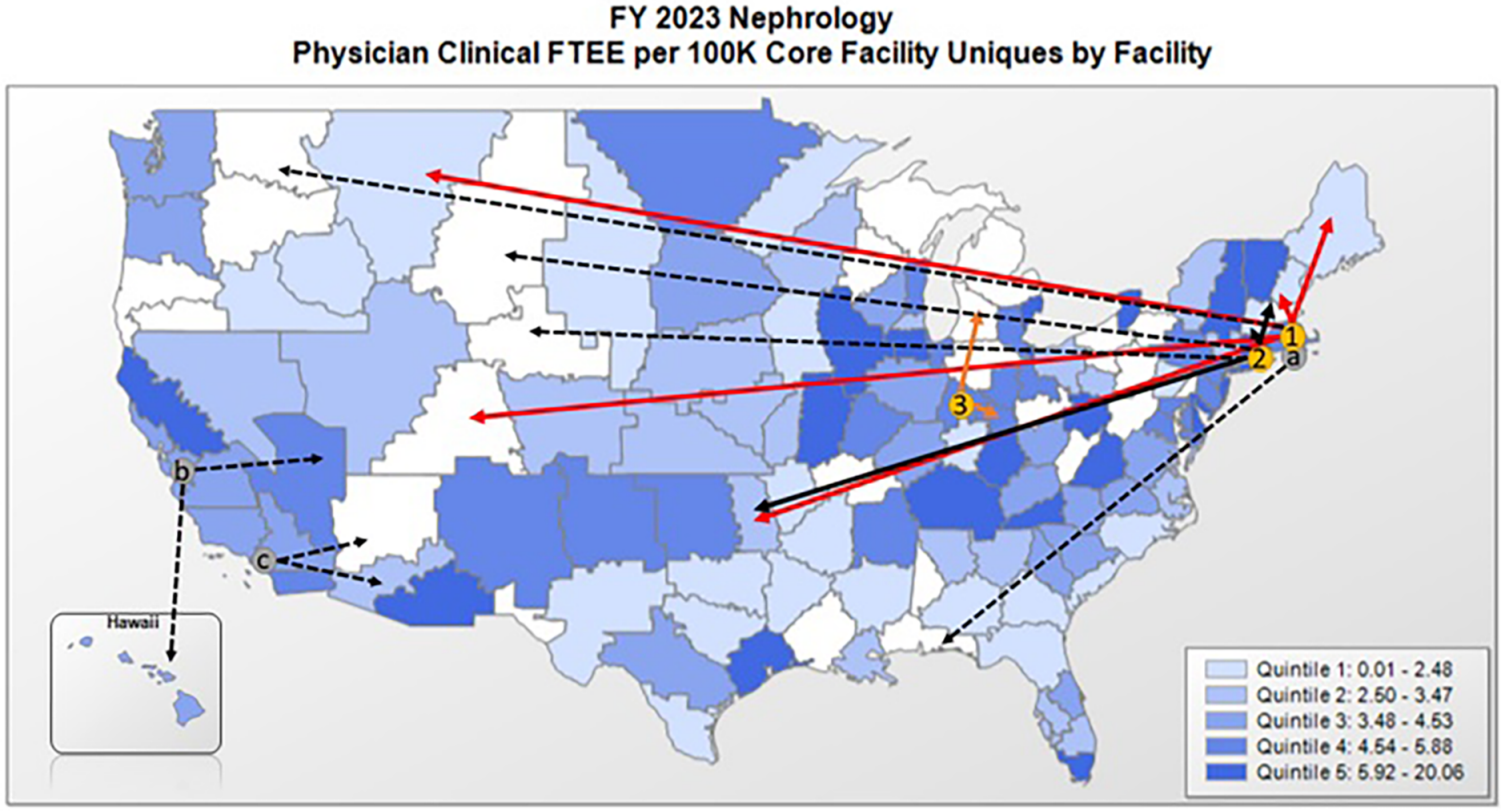
VA-based nephrology services and services provided by the telenephrology EWI. In-person nephrology specialty care is not offered at every VA facility and rural facilities often lack a VA nephrologist or depend on a single provider. The telenephrology EWI currently consists of three telenephrology hub sites in (1) Boston, (2) Connecticut, and (3) Indianapolis that provide services to originating spoke sites (solid lines) in VISN1 (Maine and New Hampshire), VISN10 (Chillicothe, OH), and VISN19 (Muskogee, OK, Grand Junction, CO, and Fort Harrison, MT). (**a**) A hub in Providence, RI will open in July of FY23 and negotiations are currently underway to expand the EWI in FY24 and FY25 to include west coast hubs in (**b**) Palo Alto, CA and (**c**) Long Beach, CA, with an aim to reach additional originating sites (dashed arrows).

To address the need for rural nephrologists, our team developed a tele-nephrology program using a hub and spoke design (Figure 1). This model leveraged nephrology specialists located at VA healthcare systems in Boston and Connecticut to consult in VA spoke sites in Maine, New Hampshire, Oklahoma, Colorado, and Montana. Spoke sites were encouraged to actively participate in care by identifying a mid-level provider to be a local renal champion. This physician assistant or nurse practitioner (APRN) received support and on-the-job training from the hub nephrologists to triage consults and to deal with emergencies and follow-up care. Veterans with CKD or rHTN initially presented to their local VA-health care center or a community-based outpatient clinic (CBOC) for their video-based tele-nephrology visits. Laboratory testing and imaging studies were arranged prior to the visit or were performed during the visit for Veterans with large travel distances. Telehealth technicians at the spoke site performed vital signs and ensured that connection with the tele-nephrologist was established and of high quality. Nephrologists at the hub site had full access to the Veteran's local electronic medical record (EMR) and recorded their findings and recommendations in the spoke site EMR. When necessary, medications were prescribed or adjusted by the tele-nephrologist and orders could be placed for imaging or other studies. Findings were communicated to local primary care providers using the EMR or Microsoft Teams. All care was coordinated by establishing a tele-nephrology organizing center (TNOC) located at the hub sites.

The purpose of this evaluation was to examine perceptions and experiences with this tele-nephrology program among spoke site staff and clinicians using the Reach, Effectiveness, Adoption, Implementation, and Maintenance (RE-AIM) framework to guide our understanding of tele-nephrology implementation.

## Material and methods

### Study design

To better understand barriers and facilitators to tele-nephrology hub and spoke implementation practices, we conducted semi-structured interviews with fifteen clinicians at five tele-nephrology spoke sites, including VA Maine, Manchester VA, Montana VA Healthcare System, Eastern Oklahoma VA Healthcare System, and VA Western Colorado Healthcare System. We used the RE-AIM framework to develop our interview guide. To recruit interviewees, we invited every provider involved in the telenephrology program at the spoke sites to participate. The participants included the local renal champions (*n* = 5), who were all nurse practitioners, nurses, scheduling staff and physician leaders. Interviews were 30–45 min in length and were conducted using Microsoft Teams. Qualitative data was initially analyzed using open thematic analysis to explore the data, and following that, the data were structured using the RE-AIM framework (12). Our results below are presented according to each major RE-AIM dimension.

### Qualitative interviews

The primary author, a health services researcher, conducted all of the interviews. At the start of the interview, the interviewer reviewed the information about the study. The semi-structured interview guide was drafted by Dr. Mattocks using the RE-AIM framework and approved by Drs. Bonegio and Moore. It assessed attitudes regarding telemedicine and its adoption to provide specialist nephrology care to rural Veterans, transitions in care and treatment between primary care providers, community-based nephrologists, and VA tele-nephrologists, the impact of tele-nephrology on the delivery of care to rural Veterans, and potential challenges to the maintenance of the tele-nephrology program. All interviews were transcribed using Microsoft Teams and entered into ATLAS.ti qualitative analysis software for analysis. Transcripts were analyzed qualitatively using thematic analysis, a systematic approach of identifying important themes in delivering remote nephrology care. Using the RE-AIM framework as a guide, we first conducted open coding in which two investigators (KM and LW) identified key concepts emerging from the language used by participants and assigned codes (descriptive phrases) to segments of text. These codes were used to create a top-level codebook that was applied to all qualitative data. At all stages, coding was performed and discussed by two investigators, and the codebook was refined until agreement was reached. Themes that emerged in the interviews were examined for similarities and differences in perspectives in a process known as constant comparison analysis. Subsequently, prominent themes and quotes exemplifying each were presented to the research team and refined.

## Results

### Participant characteristics

Our sample included 14 providers from 5 VA medical centers across the United States. A majority of the providers represented in the interviews were from VA Maine (29%) or the Eastern Oklahoma VA Healthcare System (29%). Providers interviewed included the local renal champions (28%) and physicians (21%). A majority of the participants were female (71%) and white/Caucasian (71%). The average age of participants was 48 and the average length of VA employment was 9 years (Table 1).

**Table 1:** Demographics of participants (*n *= 14).

Characteristics	Frequency	(%)
VA Location		
Eastern OK	4	(29)
Grand Junction (CO)	3	(21)
Maine	4	(29)
Manchester (NH)	1	(7)
Montana	2	(14)
Degree		
BS/BA	2	(14)
MBA	2	(14)
NP	2	(14)
DNP	2	(14)
MD	3	(21)
PhD	1	(7)
Other	2	(14)
Sex		
Male	4	(29)
Female	10	(71)
Race/Ethnicity		
Black	1	(7)
White	12	(86)
Asian	1	(7)
Average years at the VA	9	
Average age	48	

Five major themes arose that aligned with the RE-AIM framework that represent the success of the intervention at each site: (1) (Reach) Active engagement of a centralized clinical champion was a key factor in early success of tele-nephrology program; (2) (Effectiveness) Transition from community-based nephrology to VA tele-nephrology was heralded as the most meaningful indicator of the effectiveness of the intervention; (3) (Adoption) Effective adoption strategies included bi-weekly training with Hub nephrology staff and engagement of nurse practitioners; (4) (Implementation) Meeting the needs of Veterans through proper staffing during tele-nephrology examinations was a key priority in facility program implementation; and (5) (Maintenance) Growing reliance on Hub nephrologists may give rise to insufficient availability of nephrology appointments in some Spoke sites. Each of these RE-AIM themes is described in detail below.

#### Reach: active engagement of a centralized clinical champion was a key factor in early success of the tele-nephrology program

The active engagement of the primary Hub nephrologist as a clinical champion for tele-nephrology was cited by numerous spoke providers as the most important reason that the intervention had been successful to date. Staff noted that the Hub nephrologist was easy to contact with questions and staff perceived that the Veterans were comfortable and easily engaged with the primary Hub nephrologist. For example, a nurse in Montana noted:

*Our communication with the Hub nephrologist is one of the biggest positives. (The Hub nephrologist) was willing to meet with the primary care providers at one of their meetings to introduce themselves, talk about what they do, and answer any questions. I think that went a long way. Providers like to know the other providers*.

Similarly, a nurse in Oklahoma added:

*(The Hub nephrologist) will take his time and teach you and make sure you understand. And I know that he does that for the patients too. I've even called him up with the patient in my room, and he's answered*.

This sentiment was echoed by another staff member in Eastern Oklahoma:

*(The Hub nephrologist) had a day or a time slot that was dedicated to Muskogee, but he never abided by that. He was always available to us. I found that to be helpful*.

A nurse from Maine concurred:

*Those folks really have shown us that is tele-nephrology can be a model for other clinics. We basically copied a lot of the systems that they use for the Muskogee clinic for Maine. And it worked beautifully. Even if we hire a full-time nephrologist, I don't really want to stop tele-nephrology*.

#### Effectiveness: transition from community-based nephrology to VA tele-nephrology was heralded as the most meaningful indicator of effectiveness of intervention

When sites were queried about how to assess tele-nephrology program effectiveness or consider outcome measures, there was substantial variability in how sites measured program effectiveness. For several sites, the most important measure of success was moving Veterans from community-based nephrology to VA tele-nephrology. A physician from Maine noted:

*If you wanted to ask me what I want for outcome measures, I would look to see if we've been successful in seeing an increase in VVC (VA-Video Connect) visits and CVT (Clinical Video Telehealth) visits and a decrease in visits to community care visits*.

A nurse practitioner from Eastern Oklahoma concurred:

*We called a lot of the people who were out in the community, not the ones that were on dialysis, but the ones that were mostly stable. I explained to them the reason I was calling is that we are pretty much offering the same thing in the VA as what you're getting outside from community provider, and this VA provider can see your history, and you will not have to start fresh every time you go. We were able to pull back at least three out of every five Veterans in the community*.

Others noted the importance of outcomes related to hospital readmissions and patient satisfaction. A physician in Maine noted:

*I would love to know specifically about community hospital admissions. I think that would be really valuable to see if we're doing better at keeping patients out of the hospital using virtual care*.

Similarly, a nurse practitioner in Oklahoma added:

*So overall, just patient satisfaction, maybe the hospital readmission rates. That would be something that would be interesting to see. And then how long we can keep Veterans off dialysis*.

#### Adoption: effective adoption strategies included bi-weekly training with hub nephrology staff and engagement of local renal champions

Intensive bi-weekly training sessions were a critical element identified during interviews to help launch the tele-nephrology programs at each site. A nurse practitioner from Grand Junction noted:

*We were meeting every two weeks, but now we've decreased that to just once a month. (The Hub team) has been really good in coordinating follow-up if we've had a problem that has been brought up during the course of those meetings. So that is a huge positive. And I think just having those regular meetings have been very beneficial and you feel more like you are part of a team*.

Similarly, a staff member in Eastern Oklahoma added:

*During our first meetings with the Hub nephrologist we talked about how to order things in the VA system. How do you place a consult for infusions, for example? And when we get that all settled, we talked about what's up and coming. Are we looking at hiring in a nephrologist? Do we need to add more support to what's already supporting you? And then the telehealth techs go over trouble with their scheduling system. There were also a couple of calls about specific Veterans*.

*Local renal champions* were also identified as critical elements to support the tele-nephrology program, especially between visits with Hub nephrologists. A nurse from Montana noted:

*We were funded for a 0.5 FTE nurse practitioner to help with tele-nephrology. This person will do face-to-face follow-ups after (the Hub nephrologist) sees the Veteran. I think that will be a good addition for sure, because the more return to clinics appointments that provider can see, it opens up more slots for new consults (for the Hub nephrologist)*.

A nurse practitioner from Grand Junction, Colorado concurred:

*What (the Hub nephrologist) has worked towards is having someone on the ground as a liaison has been great. We've got the expertise of that nephrologist, but we also have somebody with feet on the ground that can take on those face-to- face visits in the event that they need to have a return to clinic sooner*.

#### Implementation: meeting the needs of veterans through proper staffing during tele-nephrology examinations was a key priority in facility program implementation

Several important areas of implementation emerged during the interviews. The first was the focus on ensuring that the Veteran was comfortable during the virtual visit and that the Veteran had a means to understand what was happening during the visit. A nurse practitioner from Colorado explained:

*When a Veteran came in, I would explain the process to them and stay with them until the Hub nephrologist is actually on the computer and talking to them. Then I leave the room, they can take their masks off so it's much more comfortable for them. The Hub nephrologists can see their faces. He turns his screen around and he explains everything and shows them everything. It's very educational for them and I leave a pad and paper next to the computer so they can write everything down*.

Similarly, a nurse practitioner from Eastern Oklahoma agreed:

*Sometimes, you know when you go in, you forget what the doctor said. So now we do have a little paper that if the patient seems to be not remembering everything that the doctor is telling them, we'll write those down and then hand it to the veteran when they leave. That way it'll help them when they go home. They're like, what did he say? And you know, they'll have our direct phone number to call and then that way we can, you know, tell them whatever they had questions about*.

Other implementation concerns focused on ensuring the appropriate staffing was available during the virtual visits. One Montana nurse noted:

*When we first started, we started out with RN staff being basically the telehealth techs and that was just kind of to make sure things were taken care of and the assessments were good and all that sort of thing. They've now become a little bit more relaxed on that. The telehealth techs make the situation as comfortable as they can. Usually getting through that first visit kind of eases everybody's anxieties about it*.

A nurse in Maine concurred:

*The Hub nephrologists felt that they needed a higher-level licensed person with the patients and so that limited our options because I only have a telehealth clinical technician (TCT) on staff. But I think what we've learned after a few weeks or maybe months was that they really didn't need a nurse to be present with the Veterans. Most of what they were doing was well within the job description of the TCT and that would then open up our ability to use all these other clinics. So what we did is we built additional clinics and said let's just start having TCT present then*.

#### Maintenance: growing reliance on Hub nephrologists may give rise to insufficient availability of nephrology appointments in some spoke sites

The success of the tele-nephrology program has given rise to potential barriers related to access. Several of the spoke sites noted that since they had directed many non-urgent referrals to Hub nephrologists, their sites needed additional tele-nephrology appointment times to accommodate the patient volume that had been transferred from community providers. A nurse in Montana noted:

*It is difficult that the Hub nephrologist only gives Montana VA 4 h a week. We would love to have more provider time because of course once they establish with the Hub nephrologist, they have to come back and those Veterans take up those appointments as well*.

Similarly, a nurse from VA Maine noted that since they had steered their community nephrology patients to tele-nephrology care, there was only one morning each week that the nephrologist could accommodate this growing cohort of Veterans:

*I think our strategy is really that we're trying to pull back on having to use community care at all. We're trying to be able to reach the distant parts of Maine by using the CVT clinics. The Hub nephrologist works for Maine on Tuesday mornings. He does a lot of work with other states as well, but in terms of Maine, his focus is going to be northern Maine*.

A nurse in eastern Oklahoma noted that it became important to gain access to the Hub nephrologist's schedule so that she could have a better sense of how far out he was booking patients:

*I had to get access to the scheduling program just so I could see how far out the Hub nephrologist was scheduled. And that way I wasn't constantly trying to get a hold of someone to find out his schedule. And if they didn't answer, then I'd just have to tell the veteran someone will call you. I know that they don't like that answer because if they call and then they missed the call the consult gets cancelled*.

Two of the five spoke sites hired nephrologists since the tele-nephrology program launched, but given VA rules related to drive time, Hub tele-nephrologists were still needed to see the Veterans whose drive times to the onsite nephrologist exceeded 60 min. A nurse practitioner in eastern Oklahoma noted:

*Our needs have shifted a little bit because we hired a nephrologist in Tulsa near where many patients live, so in Muskogee and Tulsa within that 60-minute radius, we now have enough providers between nurse practitioners with the Hub and the nephrologist to give that face-to-face visit. Now the area we do not have coverage seems to be the outliers from outside of that 60-minute coverage radius. Those areas still need coverage, and the Hub can really help in those areas*.

## Discussion

To the best of our knowledge, this is the first study that has evaluated the implementation of a tele-nephrology program in the VA. In the past five years, the total amount that VA has spent on community care has steadily increased, from $7.9 billion in 2014 to $17.6 billion in 2021 (13). As the costs for community care have risen, the share of the VA budget that goes toward community care has also increased. In 2014, community care accounted for approximately 12 percent of VA spending. VA's fiscal year 2024 budget request anticipated that community care would increase to 25 percent of the agency's medical care budget primarily due to increased program utilization (14). As a result of this substantial increase in outlays for community care, VA has been examining innovative virtual solutions to reduce costs and keep Veterans in the VA system for care.

A significant finding from our study was the importance of an engaged clinical champion to guide the tele-nephrology program *development*. Clinical champions are often described as possessing passion, enthusiasm, and drive to create change (15), and are frequently characterized as being effective communicators (16). Furthermore, an understanding of organizational structure and culture allows a clinical champion to leverage appropriate relationships for creating change (17). In the current study, the lead Hub nephrologist contributed to the overall success of the tele-nephrology implementation with open and frequent communication, effective and ongoing training, willingness to work outside allotted hours for spoke sites and the ease at which he worked with Veterans during virtual care sessions. While these qualities have contributed to a successful tele-nephrology development, the absence of these qualities calls into question the implementation of similar VA virtual programs for other specialties.

Our study also documented the importance of local renal champion such as NP, APRNs and PAs to see patients for non-urgent needs between tele-nephrology visits. These local champions help to triage consult urgency and address urgent medical needs when tele-nephrology in not immediately available. Renal disease is accompanied by significant risk for acute blood pressure and electrolyte abnormalities; therefore, it is critical to have midlevel local champion available to manage such issues. In some sites, these midlevel providers are also able to do follow-up care, which helps to maintain access to the Hub tele-nephrologist for new consults. Future studies will assess the impact of local renal champions on overall access and the complexity of patients managed by the tele-nephrology team. Additionally, quality improvement studies can seek to understand how to implement telenephrology teams with renal champions as additional sites as tele-nephrology initiatives are spread to other rural sites across VA.

Importantly, our study points to important access challenges that may arise within VA as community care is shifted back to VA for cost containment purposes. For years, VA has been grappling with “make vs. buy” decisions regarding what care to keep in VA vs. what care to be sent to the community (18, 19). Many factors contribute to this decision, including facility size and complexity, urban/rural setting, and the paucity of sub-specialty medical providers outside large urban areas. However, the COVID-19 pandemic prompted an abrupt shift from in-person to virtual patient encounters within the VA, as well as across the country and world. To minimize COVID-19 risk, VA explicitly encouraged providers to substitute virtual care, including phone or video visits, for in-person patient visits when feasible (20–22). Consequently, VA care that was previously only provided face-to-face is now possible virtually, increasing access for Veterans who do not live near either VA or non-VA community providers. With this knowledge, VA has increasingly begun to offer VA virtual care through telehealth hubs (23), but in order for this shift to be successful, VA must have a sufficient number of virtual providers so as not to create new access problems for virtual care.

Rural Veterans face a geographic barrier to receiving nephrology care. They often qualify for community-based care by being more than 60 min drive from a VA facility or VA nephrologist but struggle to get appointments with a community-based nephrologist, who also often practice from urban/suburban areas. Tele-nephrology offers a good solution and could be incorporated into VA care that include a local nephrologist to improve access.

Several important limitations of this evaluation are worth noting. First, we had a relatively small number of provider and staff participants in the current study, though this was reflective of the relatively small number of staff across the spoke sites engaged in the implementation itself. Secondly, spoke sites varied in the length of time that they had been engaged in the intervention, with one study site implementing tele-nephrology for more than two years and another site less than a year. Therefore, some sites were further along in the “maintenance” phase of the intervention than other sites, and thus had more established tele-nephrology programs than others.

## Data Availability

The raw data supporting the conclusions of this article will be made available by the authors, without undue reservation.
